# Using arterial spin labeling to examine mood states in youth

**DOI:** 10.1002/brb3.339

**Published:** 2015-04-20

**Authors:** Nina Mikita, Mitul A Mehta, Fernando O Zelaya, Argyris Stringaris

**Affiliations:** 1 Department of Child and Adolescent Psychiatry, King's College London, Institute of Psychiatry, Psychology & NeuroscienceLondon, UK; 2 Centre for Neuroimaging Sciences, King's College London, Institute of Psychiatry, Psychology & NeuroscienceLondon, UK

**Keywords:** Adolescents, happiness, mood, perfusion magnetic resonance imaging

## Abstract

**Introduction:**

Little is known about the neural correlates of mood states and the specific physiological changes associated with their valence and duration, especially in young people. Arterial spin labeling (ASL) imaging is particularly well-suited to study sustained cerebral states in young people, due to its robustness to low-frequency drift, excellent interscan reliability, and noninvasiveness. Yet, it has so far been underutilized for understanding the neural mechanisms underlying mood states in youth.

**Methods:**

In this exploratory study, 21 healthy adolescents aged 16 to 18 took part in a mood induction experiment. Neutral, sad, and happy mood states were induced using film clips and explicit instructions. An ASL scan was obtained following presentation of each film clip.

**Results:**

Mood induction led to robust changes in self-reported mood ratings. Compared to neutral, sad mood was associated with increased regional cerebral blood flow (rCBF) in the left middle frontal gyrus and anterior prefrontal cortex, and decreased rCBF in the right middle frontal gyrus and the inferior parietal lobule. A decrease in self-reported mood from neutral to sad condition was associated with increased rCBF in the precuneus. Happy mood was associated with increased rCBF in medial frontal and cingulate gyri, the subgenual anterior cingulate cortex, and ventral striatum, and decreased rCBF in the inferior parietal lobule. The level of current self-reported depressive symptoms was negatively associated with rCBF change in the cerebellum and lingual gyrus following both sad and happy mood inductions.

**Conclusions:**

Arterial spin labeling is sensitive to experimentally induced mood changes in healthy young people. The effects of happy mood on rCBF patterns were generally stronger than the effects of sad mood.

## Introduction

Little is known about the neural correlates of mood states and the specific physiological changes associated with their valence and duration, especially in young people. Here, we investigate these correlates using a magnetic resonance imaging (MRI) technique known as arterial spin labeling (ASL; Detre and Alsop 1999) following the induction of neutral, sad, and happy moods in a group of healthy adolescents. We exploit the phenomenon of neurovascular coupling (Attwell et al. 2010) by measuring the changes in regional cerebral blood flow (rCBF) that accompany the onset and maintenance of specific mood states.

One reason for limited research into the neural substrates of mood is methodological. Experimental designs using functional magnetic resonance imaging (fMRI) typically involve stimulus change measured in the timescale of seconds (Matthews and Jezzard 2004). It is also important to understand what underlies the persistence of mood states over sufficiently long periods given that the diagnosis and monitoring of patients requires measurement of psychopathology in the order of several hours to days and weeks. Time-series fMRI data using the blood-oxygen-level-dependent (BOLD) contrast are sensitive to a low-frequency drift, and thus less reliable when investigating neural activation changes over periods lasting longer than seconds (Smith et al. 1999). Electroencephalography (EEG), used to track brain activation changes over time, is not directly sensitive to subcortical neural activity (Kennett 2012) and has poor spatial resolution, making it less suited for investigation of limbic regions that are implicated in mood. Existing evidence on the neurophysiological correlates of mood states comes mainly from positron emission tomography (PET) studies that measured rCBF in participants experiencing experimentally induced sadness or happiness (e.g., George et al. 1996; Mayberg et al. 1999; Liotti et al. 2002; Keightley et al. 2003). However, the reliance of PET on radio-labeled compounds makes this method unsuitable when studying children and adolescents. This is unfortunate as mood disorders such as depression have their origins in adolescence, with a sharp increase in prevalence reported with the onset of puberty (Maughan et al. 2013). Recent studies have also provided evidence for a link between adolescent depression and psychopathology later in life (Thapar et al. 2012). Therefore, discovering the physiological patterns associated with mood states in adolescence is particularly important.

Arterial spin labeling appears especially well-suited to studying the neural signatures of different mood states and their specific features, such as duration and intensity. First, as blood flow contrast is generated by the pair-wise subtraction of successively acquired pairs of images (see Materials and Methods section), the data are substantially free of low-frequency sources of contamination such as physiological noise and scanner drift, and less sensitive to subject movement (Smith et al. 1999; Aguirre et al. 2002; Detre and Wang 2002; Howard et al. 2011). Second, the ASL pulse sequence acquisition parameters are tailored to maximize blood flow information from tissue capillaries and the data are therefore a more faithful signature of functionally driven changes in neurovascular coupling (see Materials and Methods section). Unlike PET, ASL is noninvasive and has been previously used in children and newborns (e.g., Wang et al. 2003; Biagi et al. 2007). Arterial spin labeling was already shown to successfully distinguish between states of depression in adults (Lui et al. 2009) and between adolescents with and without depression (Ho et al. 2013). In addition, its excellent interscan reliability (Hermes et al. 2007; Hodkinson et al. 2013) makes it suitable for monitoring treatment effects.

Mood induction is an established experimental manipulation where the participant's mood state is temporarily changed. It can be used in within-subject designs, where each participant serves as his or her own control for different mood states, and specific characteristics of the stimulus, such as intensity, can be manipulated. Mood induction is a more tractable method for an experimental study compared to studying patients who are already depressed as it is not confounded by the effects of illness and considerable between-patients heterogeneity (e.g., disorder severity, duration, number of previous episodes, comorbidity, and medication use).

Despite the above advantages, to our knowledge, ASL has only been used once with mood induction (Gillihan et al. 2010), in an adult sample. To test the feasibility of examining mood states in youth using ASL, we investigated brain perfusion patterns involved in mood changes in a sample of healthy adolescents. We used film clips combined with mood elaboration instructions, a method found to be the most reliable in inducing mood (Westermann et al. 1996) and compared sad and happy mood conditions against the neutral. The inclusion of happy condition is based on its clinical relevance to depression, a disorder characterized not only by the predominance of sad mood, but also the absence or inability to perceive positive emotions (APA 2013). fMRI studies show that adults with major depressive disorder (MDD) display a dampened neural activation to positive stimuli relative to healthy controls (Epstein et al. 2006). It is therefore theoretically important to study the neural correlates of sad and happy moods together, as dysregulation of both these mood states is relevant to depression.

We hypothesized that the mood induction procedure will lead to significant changes in self-reported mood, and that it will generate rCBF changes in areas implicated in mood processing. We used unbiased, voxel-wise, whole-brain analyses as well as predefined, bilateral regions of interest (ROIs): the amygdala, subgenual anterior cingulate cortex (sgACC), dorsolateral prefrontal cortex (dlPFC), ventromedial prefrontal cortex (vmPFC), and the ventral striatum. We expected higher amygdala activation in response to emotional (sad or happy) than neutral conditions, based on its role in encoding emotional significance. We hypothesized that the sgACC would show higher activation following sad versus neutral mood induction, based on previous PET mood induction studies in adults (Mayberg et al. 1999; Liotti et al. 2002; Keightley et al. 2003) and sgACC hyperactivity in patients with depression (Drevets et al. 2008b), recently replicated in adolescents with depression using ASL (Ho et al. 2013). Prefrontal ROIs were chosen based on their role in regulating limbic activity (e.g., Davidson 2002; Drevets et al. 2008a); therefore we expected these regions to be more activated in emotional (sad or happy) conditions compared to neutral. We also hypothesized increased rCBF in the ventral striatum following happy versus neutral mood induction, based on the relation between ventral striatal activity and euphoria in healthy adults (Drevets et al. 2001). Finally, we explored whether the results are dependent on existing depressive symptoms.

## Materials and Methods

### Participants

Twenty-two healthy adolescents aged 16 to 18 (10 males, 12 females) were recruited via adverts on social media websites and Internet forums for teenagers. In addition, one parent/carer of each participant completed a series of questionnaires (see below) about their child. One female participant was removed from subsequent analyses due to persistently high levels of anxiety while in the scanner, leaving a final sample of 21 participants. All participants were right-handed as measured by the Edinburgh Handedness Inventory (Oldfield 1971). The adolescent participants did not have any serious medical, behavioral, or emotional conditions, had no history of head injuries by self-report, and did not report any contraindication to MRI. Written informed consent was obtained from all participants. This study was approved by the Psychiatry, Nursing & Midwifery Research Ethics Subcommittee at King's College London (PNM/12/13-44).

### Questionnaires

Participants were screened for the presence of behavioral and emotional difficulties before taking part in the study with a series of self- and parent-reported questionnaires. Mood and Feelings Questionnaire (MFQ; Costello and Angold 1988) was used to measure depressive symptoms present in the previous 2 weeks. Symptoms of trait anger and irritability in the previous 6 months were measured using the Affective Reactivity Index (ARI; Stringaris et al. 2012). Additional emotional and behavioral symptoms were measured using the Strengths and Difficulties Questionnaire (Goodman 1997). Participants scoring high on any of these measures were excluded from the study. Individual cases were discussed with an attending physician (A.S.).

### Procedure

#### Stimuli

Based on a meta-analysis of mood induction procedures (Westermann et al. 1996), emotional film clips coupled with mood elaboration were chosen as a way of inducing mood. Each film clip was approximately 4 min long and depicted the following: neutral clip – a young man describing how to clip in and out of mountain bike pedals; sad clip – a scene from Dead Poets Society (Weir, 1989) where a teenage boy finds out that his best friend committed suicide; happy – a series of stand-up comedy routines by a British comedian, Michael McIntyre. Before seeing each film clip, the participants were instructed to enter the specified mood state (as used previously by Habel et al. 2005). The instructions were as follows: “During this task, I would like you to try to become sad/happy. To help you do that, I will show you a video that most people find sad/happy”. After seeing each film clip, the participants saw a message asking them to think about how the film had made them feel (as used previously, e.g., by Furman et al. 2011). For instance in case of sad mood condition, the instructions were as follows: “Have you ever been in a similar situation? Have you ever lost a loved one and if so, how did it make you feel? How would you feel if you were in the same situation?”

All participants were shown the scanner environment and invited to lay down inside our mock scanner in order to familiarize themselves with the scanning environment and reduce the potential for drop out. After confirming that they were ready to proceed, the participants entered the MRI scanner. First, a structural MRI scan was taken. The participants then rated their mood on a scale from 0 (very sad) to 10 (very happy), followed by the neutral mood induction that served as a baseline. After having watched the film clip, participants rated their mood again. This was followed by the first ASL scan (7:15-min long, see below) during which the participants were instructed to remain still and look at the screen with the following text: “Think about how you felt when watching this neutral film clip. Please try to maintain this feeling while you're being scanned.” The procedure was then repeated for the sad and happy conditions. Mood ratings for each condition were collected immediately after the end of each film clip.

#### MR imaging

The scanning was carried out at the Centre for Neuroimaging Sciences, Institute of Psychiatry, King's College London using a General Electric MR750 3.0T scanner.

In ASL, the MRI signal of endogenous arterial blood water is used as a contrast agent to measure rCBF. The contrast is achieved by “labeling” or “tagging” a bolus of arterial blood, by inverting its magnetization in the region of the carotid arteries with an external (noninvasive) radiofrequency pulse. If two whole-volume images are rapidly acquired in succession (one with and one without labeling of arterial blood), the resultant *difference* image is proportional to the volume of blood perfused into each unit volume of tissue during the time between the labeling and the acquisition of the image. This time is typically long enough (1.5 sec) so that the contrast is derived from labeled water in the microcirculation (capillaries) and not in any of the larger arterioles. A suitable model is employed to convert the difference image into a map of rCBF in conventional physiological units of mL blood/100 g tissue/min. As stated earlier, the continuous pair-wise subtraction of labeled and nonlabeled images makes ASL suitable for tasks using longer lasting stimuli due to the low sensitivity to signal drift.

Each ASL image volume of 54 slices (3-mm thickness, no interslice gap) was acquired using a pseudocontinuous flow-driven adiabatic inversion scheme (Dai et al. 2008); TE/TR = 11.088/4901 ms, flip angle (FA) = 111°, postlabeling delay 1525 ms. Acquisition of five control and labeled pairs was done with a 3D FSE, multishot spiral stack, employing eight spiral arms for each interleave in a total of 7:15 min. Spiral k-space data were regridded to a 256 × 256 in-plane matrix prior to Fourier transformation. A single proton density scan with the same acquisition parameters was used as a reference to compute rCBF in standard units. This procedure yielded rCBF maps with a resolution of 2 × 2 × 3 mm. Enhanced fast gradient echo three-dimensional sequence was used to collect T1-weighted images, with TR = 7.312 msec, TE = 3.016 msec, inversion time 400 msec, FA = 11^0^, field of view = 270 mm, 256 × 256 matrix, 196 sagittal slices 1.2-mm thick.

### Image processing

Image processing and analyses were performed using the Statistical Parametric Mapping suite (SPM, Functional Imaging Laboratory, University College London, London UK, version 8, www.fil.ion.ucl.ac.uk/spm; RRID:nif-0000-00343). ASL images were normalized to the standard space of the Montreal Neurological Institute (MNI) by the following procedure: first, raw rCBF maps were coregistered to the high-resolution T1-weighted anatomical volume after coarse alignment of the origin of both images. Segmentation of the T1-weighted image yielded a “brain-only” binary mask which was multiplied by the coregistered rCBF map to produce an image free of extracerebral artifacts. Finally, the T1-weighted image was transformed to the T1-weighted MNI template and the transformation parameters applied to the clean rCBF maps. All normalized rCBF maps were then spatially smoothed with a 8 × 8 × 8 mm kernel.

#### ROI definition

ROIs (all bilateral) were defined using the WFUPickAtlas toolbox (RRID:nif-0000-00358; Maldjian et al. 2003) available in SPM. The sgACC was defined as Brodmann area (BA) 25 and dilated by 1 voxel. The dlPFC was generated by combining BA 9 and BA 46, and was dilated by 1 voxel. The amygdala was defined using the Automated Anatomical Labeling (AAL) library (Tzourio-Mazoyer et al. 2002). The vmPFC ROI combined bilateral medial orbital frontal and rectus regions from the AAL atlas. As the atlas does not include a predefined mask for the ventral striatum, this ROI was defined as two 8-mm spheres based on MNI coordinates (right: *x* = 9, *y* = 9, *z* = −8; left: *x* = −9, *y* = 9, *z* = −8) derived from a previous meta-analysis (Postuma and Dagher 2006) as used by Nusslock et al. (2012).

### Statistical analysis

We first examined the effectiveness of our mood induction procedure in producing stimulus-congruent mood changes using repeated-measures analyses of variance (ANOVAs) and paired-samples t-tests on self-reported mood ratings.

Subsequently, whole-brain analysis of ASL images from the three mood induction conditions was performed using a one-way, within-subjects ANOVA with gender and mean global CBF added as covariates. This was due to small, but significant changes in global CBF that occurred during the time inside the scanner [*F*_2,38_ = 8.66, *P *=* *0.001, 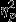  = 0.313; mean global CBF decrease from 56.2 to 53.9 mL blood/100 g tissue/min]. As this was an exploratory study and to date there is no consensus regarding statistical analysis of ASL “activation” data, we employed two different methods to indicate significance of findings at the whole-brain level. First, we used the stringent, SPM-derived significance of *P *<* *0.05 with family wise error (FWE) correction based on cluster extent. Second, we performed Monte Carlo simulations using the AlphaSim program in Resting-State fMRI Data Analysis Toolkit (Song et al. 2011) to determine the cluster size (number of voxels) needed in order to achieve a corrected *P* lower than 0.05; thresholding the statistical images with a cluster-forming threshold of *P *=* *0.01 and clustering with a cluster connection radius of 2 mm. Minimum cluster size for all individual analyses are provided in the results section. For all ROI analyses, small volume correction in SPM was used, FWE corrected at the voxel level.

We then investigated whether the amount of self-reported mood change from neutral to sad/happy correlated with the amount of change in brain perfusion patterns. To do this, we performed a multiple regression analysis with the difference in self-reported mood scores (sad or happy minus neutral) regressed against the difference between respective perfusion images (neutral subtracted from sad or happy). Gender and mean global CBF were added to the model as covariates.

The effects of depressive symptoms on brain perfusion patterns following mood induction were examined using multiple regression. Total MFQ score was added to the model as a predictor, and the “perfusion difference” image (neutral subtracted from sad or happy) as the outcome. Gender and mean global CBF were added to the model as covariates.

## Results

### Behavioral results

#### Mood ratings

**Figure 1:**
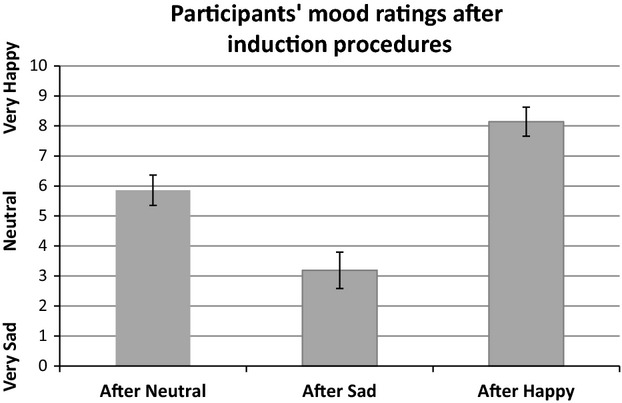
Mean mood ratings (with 95% confidence intervals) from 21 participants after watching a neutral, sad, and happy film clip in the scanner.

As illustrated in Figure1, the mood induction procedure led to significant changes in self-reported mood ratings among the participants, *F*_1.41,28.10_ = 143.48, *P *<* *0.001, 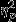 = 0.878. Compared to the neutral condition, the participants rated their mood significantly lower after seeing the sad film clip, *t*_20_ = 12.02, *P *<* *0.001, *d *=* *2.18, and significantly higher after seeing the happy clip, *t*_20_ = 8.81, *P *<* *0.001, *d *=* *2.10. The difference in ratings between the sad and happy conditions was also significant, *t*_20_ = 13.22, *P *<* *0.001, *d *=* *4.19.

#### Questionnaire data

Total SDQ scores indicated average levels of emotional and behavioral difficulties in the sample by self- (mean = 7.7, SD = 3.9) and parent-report (mean = 4.9, SD = 3.9). Anger and irritability levels were low by both self- (mean ARI = 1.6, SD = 1.5) and parent-report (mean ARI = 2.3, SD = 2.5). Lastly, there was a strong cross-informant agreement between depressive symptoms as rated by the young people themselves (mean MFQ = 6.9, SD = 6.1) and by their parents (mean MFQ = 2.8, SD = 2.8); *r *=* *0.50, *P *<* *0.05.

### Neuroimaging results

#### Sad mood

**Figure 2:**
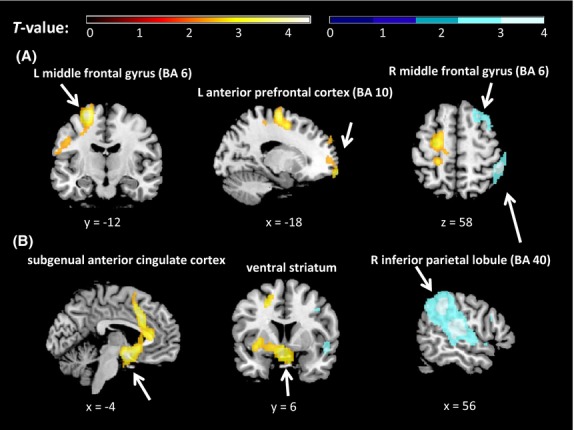
Whole-brain level ANOVA results showing regional cerebral blood flow (rCBF) levels for the contrasts (A) sad versus neutral and (B) happy versus neutral, overlaid on a T1-weighted structural brain image. Orange = increased rCBF relative to neutral, blue = decreased rCBF relative to neutral. All locations are reported in MNI coordinates. For illustration purposes, the cluster-level significance is *P *<* *0.05 (AlphaSim corrected). BA, Brodmann area; L, left; R, right.

At whole-brain level, sad mood induction led to increases in two clusters that were significant based on cluster size AlphaSim threshold, but not FWE correction (Table 2002a, Fig.2A), adjusted for the global CBF. The first, larger cluster with a peak in the left middle frontal gyrus also encompassed left postcentral gyrus. The second cluster included left medial superior frontal gyrus and BA 10. In contrast, right superior and middle frontal gyri and right inferior parietal lobule (BA 40) showed decreased rCBF after sad compared to neutral mood induction (Table 1, Fig.2A). No significant ROI results were found.

**Table 1:** Whole-brain level analysis results for (a, b) the ANOVA sad versus neutral contrast, and (c) correlation between self-reported mood ratings difference and brain perfusion maps difference for sad minus neutral mood induction conditions

Region	Side	Cluster size (voxels)	Peak MNI coordinates			*Z* score	*P* (FWE)	AlphaSim corrected^1^
			*x*	*y*	*z*			
(a) Sad > Neutral								
Middle frontal gyrus (BA 6), postcentral gyrus	L	1747	−24	−12	66	4.07	0.231	*P*_corr_ < 0.05
			−20	−2	52	2.99		
			−64	−6	16	2.87		
Medial superior frontal gyrus, anterior prefrontal cortex (BA 10)	L	744	−18	68	−14	3.40	0.752	*P*_corr_ < 0.05
			−14	58	4	3.03		
			−16	62	36	2.81		
(b) Sad < Neutral								
Superior frontal gyrus, middle frontal gyrus (BA 6)	R	550	24	16	68	3.53	0.875	*P*_corr_ < 0.05
			40	8	62	2.61		
Inferior parietal lobule (BA 40)	R	1963	50	−42	58	3.45	0.175	*P*_corr_ < 0.05
			44	−62	46	3.15		
			40	−68	40	3.11		
(c) Correlation with mood ratings								
Negative correlation								
Precuneus	R	914	8	−80	52	3.54	0.603	*P*_corr_ < 0.05
			14	−54	14	2.97		
			22	−84	48	2.87		

BA, Brodmann area; FWE, family wise error correction; L, left hemisphere; R, right hemisphere.

1 Cluster extent threshold was 477 voxels for the ANOVA and 504 voxels for correlation analyses.

We then correlated the difference in brain perfusion patterns between sad and neutral mood induction conditions with the corresponding difference in self-reported mood ratings. We found a significant negative correlation in right precuneus at whole-brain level (Table 2002c, Fig.3A), suggesting that the decrease in self-reported mood from neutral to sad condition was associated with increased perfusion changes in this region. No significant ROI results were found.

**Figure 3:**
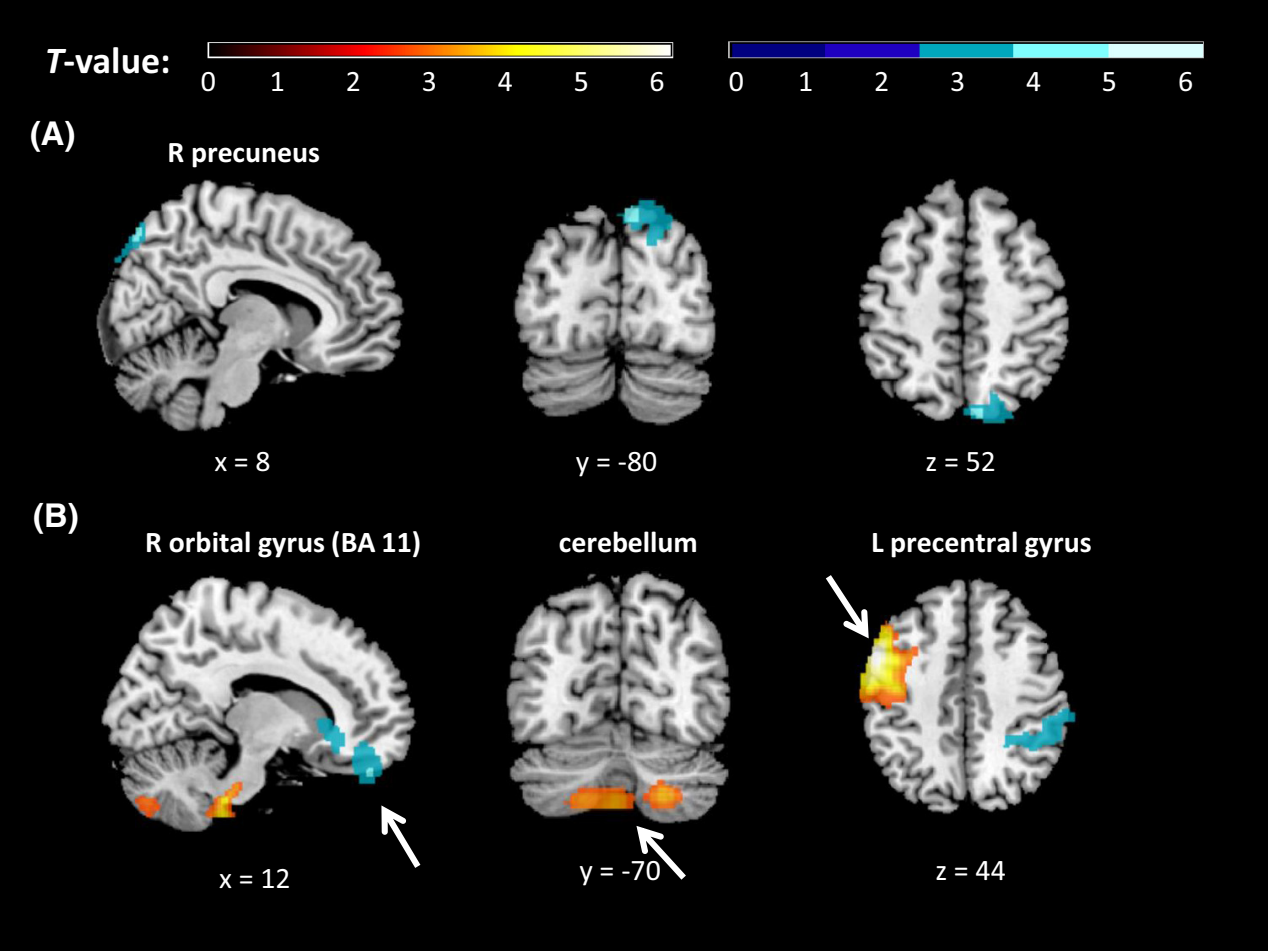
Results of whole-brain level analyses for the regressions between self-reported mood rating differences and regional cerebral blood flow (rCBF) differences for the contrasts (A) sad minus neutral, (B) happy minus neutral, overlaid on a T1-weighted structural brain image. Orange = positive correlation, blue = negative correlation. All locations are reported in MNI coordinates. For illustration purposes, the cluster-level significance is *P *<* *0.05 (AlphaSim corrected). BA, Brodmann area; L, left; R, right.

#### The role of depressive symptoms

Next, we investigated whether the magnitude of neural activation following mood induction depended on the level of current depressive symptoms. We performed a regression analysis of “perfusion difference” images (neutral condition image subtracted from sad or happy) against total MFQ scores.

**Figure 4:**
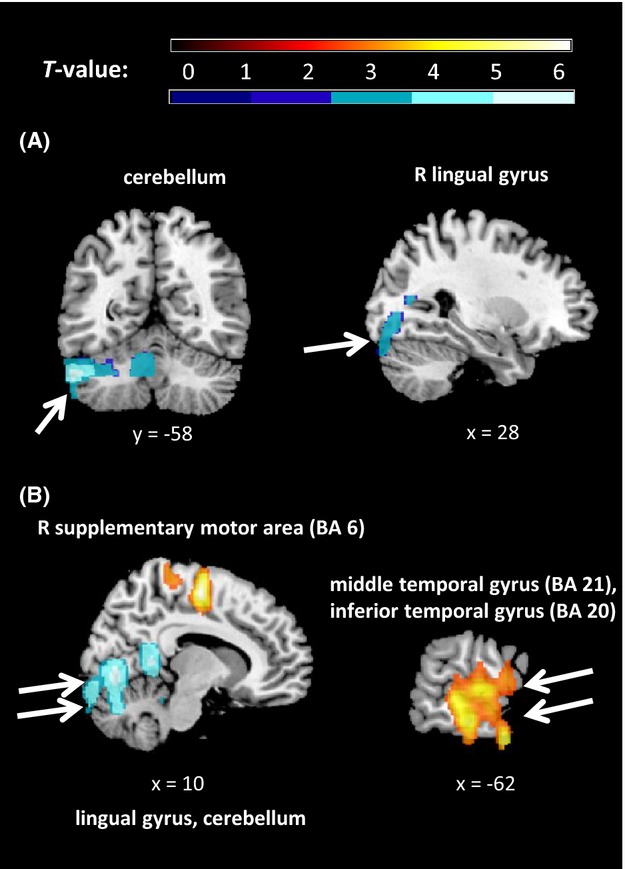
Results of whole-brain level analyses for the regressions between self-reported depressive symptoms (MFQ) and regional cerebral blood flow (rCBF) difference: (A) sad minus neutral, (B) happy minus neutral, overlaid on a T1-weighted structural brain image. Orange = positive correlation, blue = negative correlation. All locations are reported in MNI coordinates. For illustration purposes, the cluster-level significance is *P *<* *0.05 (AlphaSim corrected). BA, Brodmann area; L, left; R, right.

For sad mood condition, we found negative whole-brain level correlations between self-reported MFQ and the sad minus neutral perfusion difference. As shown in Table 2 and Figure4A, higher MFQ scores were associated with lower rCBF in bilateral cerebellum, right lingual gyrus, and right BA 18. The results were significant at the cluster size, but not FWE corrected, level. No significant ROI results were found.

**Table 2:** Correlation results between self-reported depressive symptoms (MFQ) and brain perfusion maps difference for (a) sad minus neutral, (b) happy minus neutral; all at whole-brain level

Region	Side	Cluster size (voxels)	Peak MNI coordinates			*Z* score	*P* (FWE)	AlphaSim corrected^1^
			*x*	*y*	*z*			
(a) Sad mood induction								
Negative correlation								
Cerebellum	L	1650	−54	−58	−34	4.63	0.218	*P*_corr_ < 0.05
			−24	−46	−38	3.50		
			−4	−52	−30	3.44		
Cerebellum, lingual gyrus, BA 18	R	972	28	−88	−24	3.19	0.556	*P*_corr_ < 0.05
			24	−80	0	2.90		
			24	−68	12	2.89		
(b) Happy mood induction								
Positive correlation								
SMA (BA 6)	R	2443	10	−12	58	4.36	0.104	*P*_corr_ < 0.05
			4	−32	66	3.65		
			26	−22	66	3.25		
Middle temporal gyrus (BA 21), inferior temporal gyrus (BA 20)	L	3497	−62	0	−28	3.78	0.030	*P*_corr_ < 0.05
			−62	−22	−16	3.62		
			−60	−30	−6	3.59		
Negative correlation								
Lingual gyrus, cerebellum		7503	22	−74	2	3.66	0.001	*P*_corr_ < 0.05
			32	−72	0	3.62		
			−14	−48	−36	3.46		

BA, Brodmann area; FWE, family-wise error correction; L, left hemisphere; R, right hemisphere, SMA, supplementary motor area.

1 Cluster extent threshold was 484 voxels for sad mood condition and 568 voxels for happy mood condition.

We also found whole-brain level correlations between self-reported MFQ and the happy minus neutral image difference. As can be seen in Table 2013b and Figure4B, the higher the MFQ score, the higher the rCBF after watching the happy versus neutral film clip in the supplementary motor area (SMA) and a large cluster encompassing left middle and inferior temporal gyri. In contrast, MFQ scores were negatively correlated with rCBF in a large cluster encompassing the lingual gyrus and cerebellum (Table 2013b). No significant ROI results were found.

#### Happy mood

At the whole-brain level, happy mood induction led to significant increases in rCBF in a large cluster extending from the brainstem via the cingulate gyrus to the medial frontal gyrus (see Table 2010a and Fig.2B) and a smaller cluster encompassing the subgyral areas of left parietal and frontal lobes. In contrast, a large cluster including the inferior parietal lobule showed decreased rCBF after happy compared to neutral mood induction (Table 3 and Fig.2B).

**Table 3:** Whole-brain and ROI results for (a, b) the ANOVA happy versus neutral contrast, and (c) correlation between self-reported mood ratings difference and brain perfusion maps difference for happy minus neutral mood induction conditions

Region	Side	Cluster size (voxels)	Peak MNI coordinates			*Z* score	*P* (FWE)	AlphaSim corrected^1^
			*x*	*y*	*z*			
(a) Happy > Neutral								
Whole-brain analysis								
Brainstem, cingulate gyrus (incl. BA 32), medial frontal gyrus		4461	−4	0	−16	3.89	0.009	*P*_corr_ < 0.05
			−6	16	42	3.56		
			−14	14	50	3.53		
Subgyral (parietal and frontal lobes)	L	477	−22	−36	54	3.52	0.913	*P*_corr_ < 0.05
			−28	−38	38	3.47		
			−20	−40	40	2.75		
ROI analysis								
sgACC	L	507	−4	2	−16	3.81	0.005	
Ventral striatum		271	−4	6	−12	3.38	0.010	
Amygdala		13	−16	−8	−14	2.72	0.051	
(b) Happy < Neutral								
Whole-brain analysis								
Inferior parietal lobule (BA 40)	R	6112	56	−42	38	3.51	0.002	*P*_corr_ < 0.05
			54	−24	14	3.49		
			48	−56	44	3.29		
(c) Correlation with mood ratings								
Positive Correlation								
Whole-brain analysis								
BA 8, precentral gyrus, middle frontal gyrus	L	5530	−54	6	44	4.72	0.003	*P*_corr_ < 0.05
			−30	−24	76	3.64		
			−48	22	46	3.41		
Brainstem	R	940	18	−34	−50	3.55	0.626	*P*_corr_ < 0.05
			14	−34	−42	3.54		
			0	−42	−50	3.11		
Cerebellum		1312	24	−70	−44	3.06	0.410	*P*_corr_ < 0.05
			0	−74	−44	2.95		
			−18	−70	−46	2.77		
Superior temporal gyrus	L	561	−40	−44	16	2.83	0.870	*P*_corr_ < 0.05
			−42	−48	8	2.81		
			−44	−42	28	2.79		
ROI Analysis								
Amygdala	L	78	−30	−2	−18	3.27	0.012	
dlPFC	L	1162	−54	6	42	4.71	0.002	
Negative Correlation								
Whole-brain analysis								
Orbital gyrus (BA 11), ACC, putamen	R	1241	12	48	−28	3.17	0.446	*P*_corr_ < 0.05
			8	30	−6	3.01		
			24	18	4	2.98		
Inferior parietal lobule/postcentral gyrus (BA 40)	R	1653	46	−46	56	3.05	0.269	*P*_corr_ < 0.05
			52	−34	52	2.91		
			52	−40	46	2.86		

ACC, anterior cingulate cortex; BA, Brodmann area; dlPFC, dorsolateral prefrontal cortex; FWE, family wise error correction; L, left hemisphere; R, right hemisphere; ROI, region-of-interest; sgACC, subgenual anterior cingulate cortex.

1 Cluster extent threshold was 477 voxels for the ANOVA and 540 voxels for correlation analyses.

ROI analyses revealed increased rCBF in the sgACC and ventral striatum (see Table 2010a). There was also a marginally nonsignificant finding in the amygdala (*P* = 0.051).

We then correlated the difference in brain perfusion patterns between happy and neutral mood induction conditions with the corresponding difference in self-reported mood ratings. We found a significant positive correlation in the BA 8, precentral gyrus, cerebellum, and superior temporal gyrus at whole-brain level, as well as the amygdala and dlPFC ROIs (Table 2010c and Fig.3B), suggesting that the increase in self-reported mood from neutral to happy condition was associated with increased perfusion in these regions.

By contrast, there was a negative correlation between the magnitude of self-reported mood change and rCBF between happy and neutral conditions in right inferior parietal lobule and right BA 11.

## Discussion

This was the first exploratory study to investigate the neural substrates of mood states in young people using ASL, an MRI method that is especially suited to examining prolonged neural activation. We showed that mood changes can be robustly induced in healthy adolescents using our paradigm, as evidenced by significant changes in self-reported mood without significant between-subject variance. We also found rCBF differences following sad and happy mood induction procedures compared to neutral. The amount of rCBF change was affected by the degree of induced mood change and by current depressive symptoms.

Our main finding in the sad versus neutral contrast was a change in perfusion in the middle frontal gyrus (BA 6), with increased rCBF on the left, and decreased rCBF on the right side following sad mood induction. A PET study of adult patients with depression previously showed that decreased perfusion in middle frontal gyrus can be reversed with antidepressant treatment, consistent with the involvement of this region in mood processing (Buchsbaum et al. 1997). We did not expect lateralized findings, although one previous PET study in healthy adults also found rCBF in left middle frontal gyrus to be increased, and the right decreased, when performing a cognitive task following sad versus neutral mood induction (Baker et al. 1997). Second, decreased rCBF in the inferior parietal lobule following sad versus neutral mood induction is consistent with this region's role as a component of the default mode network (DMN), a network of brain regions that are active during wakeful rest (Buckner et al. 2008). Reduction in DMN activity has been associated with self-referential processing (e.g., Sheline et al. 2009). Crucially, we also observed decreased rCBF in the inferior parietal lobule following happy mood induction, suggesting that the DMN activity was suppressed when participants actively engaged in mood elaboration regardless of mood valence. Lastly, we found a correlation between the intensity of self-reported sadness and increased rCBF in the precuneus during sad mood elaboration, consistent with the role of precuneus in the recall of episodic and self-referential memory (Cabeza and Nyberg 2000).

None of our sad mood induction findings reached the stringent, FWE-corrected significance level. There are two possible explanations. First, due to paucity of research with pediatric samples, our hypotheses were mainly based on PET mood induction studies with adults. These showed effects of both happy (Schneider et al. 1995; George et al. 1996) and sad mood induction on rCBF (Schneider et al. 1995; Keightley et al. 2003). It could be that adolescents show a weaker rCBF response to sad mood induction due to their stage of development or that there is higher variance in this response across subjects. A large-scale ASL study recently found that the trajectory of rCBF evolution undergoes dynamic changes across adolescence (Satterthwaite et al. 2014). Consistent with the developmental hypothesis, Kliegel et al. (2007) found that younger adults show lower emotional reactivity to negative mood induction compared to older adults, consistent with a weaker relation between daily stress and negative affect in younger versus older adults (Mroczek and Almeida 2004). However, the extent to which our results reflect a developmental effect remains unclear without direct comparison using matched groups. Alternatively, the healthy, never-depressed adolescents included in this study might not be as susceptible to sad mood induction as they are to the happy. Using mood induction and fMRI, Joormann and colleagues found differences in neural activation between healthy girls and girls at risk of depression in areas implicated in negative mood processing (Joormann et al. 2012). We were unable to test this hypothesis directly since the MFQ scores of our participants were all within the nondepressed range. Future studies should investigate whether rCBF reactivity to sad mood induction is higher in adolescents with than without depression. Nevertheless, even in our nondepressed sample, we did find a negative correlation between the severity of depressive symptoms (self-reported MFQ score) and rCBF in the cerebellum and lingual gyrus following both sad and happy mood inductions. This is consistent with previous fMRI research showing decreased capacity for processing happy faces in the cerebellum and lingual gyrus in adults with depression (Fu et al. 2007), an effect that was reversed by antidepressant treatment. Decreased activity in the cerebellum and lingual gyrus in response to positive stimuli was also found in euthymic patients with bipolar depression (Malhi et al. 2007) compared to healthy controls. Together with the recent finding that adolescents with depression show decreased rCBF in the cerebellum compared to healthy controls (Ho et al. 2013), our results provide some additional evidence for the involvement of the cerebellum in emotional processing (e.g., Konarski et al. 2005). We also found a positive correlation between depressive symptoms and rCBF in the SMA following happy mood induction, although the role of this region in mood processing remains unclear.

In line with our hypotheses, we found increased rCBF in the limbic regions (including the ventral striatum and a marginally not significant finding in the amygdala) following happy mood induction procedures. Moreover, there was a positive correlation between the self-reported increase in happiness and rCBF change in the left amygdala and left dlPFC. These results are consistent with the role of the frontolimbic circuitry in emotional processing, with the amygdala involved in determining the emotional content of stimuli and frontal regions modulating emotional responses. This is in keeping with mood induction fMRI findings in patients with depression, who show an opposite direction of effects compared with healthy controls. For instance, in adults with MDD, severity of depressive symptoms correlated negatively with activation in the following areas following happy, but not sad mood induction: left putamen, bilateral caudate, left nucleus accumbens, and left amygdala (Keedwell et al. 2005b). Furthermore, decreased ventral striatum activity when processing positive words in depressed versus healthy adults correlated with symptoms of anhedonia (Epstein et al. 2006), consistent with abnormalities in the reward processing system in depression. Depressed adults also show an opposite pattern of vmPFC activation following happy and sad mood induction, compared to nondepressed adults (Keedwell et al. 2005a). Finally, adolescents with depression show lower rCBF in the dlPFC at rest compared to controls, as measured by ASL (Ho et al. 2013). Future studies should investigate whether adolescents with depression show dampened rCBF responsiveness to the happy – and heightened responsiveness to sad mood induction. Notably, happy mood induction is an ethically viable way of inducing a mood state, especially in children and those at risk of a mood disorder. Given what we know about decreased positive affect in depression, happy mood induction may function as a helpful probe for detecting depression in youth.

Contrary to our hypotheses, we also found an increase in sgACC perfusion following happy, rather than sad mood induction. sgACC activation to happy (as well as sad) stimuli was previously reported in adults with treatment-resistant depression (Kumari et al. 2003); where it was suggested that the severely depressed patients responded to happy stimuli as to frustrative nonreward. However, our participants were not clinically depressed and we did not find a correlation between depressive symptoms and rCBF in the sgACC following happy mood induction in our sample. Interestingly however, healthy adults show significant functional coupling between the left amygdala and both the dlPFC and sgACC during emotion regulation (reappraisal of negative emotion; Banks et al. 2007). It is possible that coactivation of these regions in our study reflects the participants actively maintaining their happy mood following the induction procedure.

The main strength of this study is the combined use of ASL and mood induction to directly examine rCBF patterns associated with three different mood states in adolescents. Importantly, we carried out the scanning after film clip presentation, ensuring that the resulting neural activity reflected the participant's mood state and not film clip characteristics (color, brightness, or sound). This study is limited by the fixed order of mood induction conditions, used deliberately to maximize the power to detect mood-specific rCBF patterns in our sample. A larger study with a randomized order of mood induction conditions is needed to rule out the possibility of an order effect or emotional contagion. Secondly, a general limitation of mood induction methods is that self-reported mood ratings may be influenced by the participants' desire to please the examiner.

## Conclusion

This study offers a crucial starting point for the investigation of mood states, using methodology that bypasses the limitations of conventional fMRI. Although important challenges remain (Savitz et al. 2013), studying the tonic activation of neural networks involved in mood processing is likely to have important clinical implications for disorders characterized by persistently sad or happy mood, such as unipolar and bipolar depression, across development.
